# Reactive Oxygen Species Homeostasis Regulates Pistil Development and Pollination in *Salix linearistipularis*

**DOI:** 10.3390/plants15010168

**Published:** 2026-01-05

**Authors:** Xueting Guan, Chaoning Zhao, Junjie Song, Jiaqi Shi, Bello Hassan Jakada, Gege Dou, Xingguo Lan, Shurong Ma

**Affiliations:** Key Laboratory of Saline-Alkali Vegetation Ecology Restoration, Ministry of Education, College of Life Sciences, Northeast Forestry University, Harbin 150040, China; guanxt@nefu.edu.cn (X.G.); zhaochaoning@nefu.edu.cn (C.Z.); songjunjie@nefu.edu.cn (J.S.); jiaqi.shi@nefu.edu.cn (J.S.); bello.jakada@nefu.edu.cn (B.H.J.); dougege@nefu.edu.cn (G.D.)

**Keywords:** *Salix linearistipularis*, ROS, pistil, pollination, pollen tube germination

## Abstract

During the development of the gametophyte in angiosperms, a series of processes occurs, including pollination, pollen recognition, adhesion, hydration, germination, pollen tube growth, and the guidance of the pollen tube toward the ovule for the delivery of sperm cells to the female gametophyte. These processes require a substantial energy supply, which is provided by cellular respiration in the plant. Throughout this sequence, the generation of reactive oxygen species (ROS) is concomitantly observed. At present, the mechanisms underlying ROS production remain incompletely understood, especially in plant trees such as *Salix linearistipularis*. In this study, pistils of *S. linearistipularis* were used as experimental materials, and pistils were divided according to their development into three stages—S1, S2, and S3. Transcriptome sequencing (RNA-Seq) was performed for the three developmental stages, and the results indicated that metabolic pathways associated with oxidoreductase activity were highly significant during pistil development in *S. linearistipularis*. During pistil development, the levels of ROS accumulated rapidly. After pollination, with the adhesion and germination of pollen, the levels of ROS decreased significantly. Moreover, bidirectional regulation of ROS levels revealed that treatment with ROS inducers and scavengers led to increased and decreased ROS accumulation, which were accompanied by the inhibition and promotion of pollen tube number and length. These two opposite results indicate that ROS are the key factor regulating pistil development and pollen tube germination in *S. linearistipularis*.

## 1. Introduction

In flowering of tree plants, the stigma of the pistil plays a critical selective barrier role by inhibiting the germination of non-compatible (especially genetically similar) pollen and preventing fertilization of the ovule, thereby avoiding the formation of defective or genetically maladaptive embryos [1]. The sperm of male gametophyte are transported to female gametophyte through pollen tubes. This process includes pollen adhesion, hydration, germination, tip growth of the pollen tube, and pollen tube rupture [2,3]. The oxidative environment produced by the female gametophyte promotes the growth of the pollen tube [4], but unregulated generation of reactive oxygen species (ROS) on the stigma surface, such as H_2_O_2_, may inhibit pollen hydration or induce oxidative damage. Moderate application of ROS inhibitors before pollination can reduce pollen death caused by ROS and increase the number of viable pollen tubes. However, when ROS levels fall below the normal range, it will also lead to the inhibition of pollen tube growth [5]. Wang et al. found that reducing the ROS levels on the stigma of *Fraxinus mandshurica* significantly increased the number of pollen adhesion and germination [6]. However, in the *Arabidopsis thaliana* rbohH rbohJ double mutant, reduced ROS levels resulted in severe defects in pollen tube tip growth [7]. Suppressing the expression of NADPH oxidase (also known as NOXs) in *Nicotiana tabacum* pollen markedly inhibits pollen tube growth, while exogenous application of H_2_O_2_ could specifically reverse this inhibitory effect [8]. These findings collectively reveal that ROS originating from the pollen tube itself is essential for maintaining normal pollen tube growth. Therefore, changes in the ROS microenvironment are a key component in coordinating the pollen–stigma interaction. Furthermore, the regulation of ROS balance is not only essential for pollen germination but also necessary for the growth of pollen tube tips and the rupture of pollen tubes. Pollen germination that occurs on the stigma of the pistil, pollen tube tip growth, which is part of the transport phase, takes place within the style, and pollen tube rupture occurs during fertilization within the ovule. When the pollen tube reaches the ovule, high levels of ROS induce pollen tube rupture and sperm release [9]. This indicates that the differential roles of ROS in plant reproduction, dependent on their site of production within the cell and the developmental stage, may exert opposite functions.

Reactive oxygen species (ROS) is a collective term referring to a series of single-electron reduction products of oxygen, including hydrogen peroxide (H_2_O_2_), peroxyl radical (ROO^−^), superoxide anion radical (O_2_^−^), hydroxyl radical (OH·), alkoxyl radical (RO^−^), and singlet oxygen (^1^O_2_) [10,11]. When accumulated at abnormally high levels, ROS act as cytotoxic molecules in plants [12]. However, under normal physiological concentrations, it can promote plant growth and development [13]. In plant cells, organelles such as mitochondria, chloroplasts, and peroxisomes are the primary sites of ROS generation [14]. Both the normal growth metabolism of plants and external disturbances can induce the generation of ROS [15,16]. Plants can detect changes in ROS levels and transmit signals throughout the organism. Plant antioxidants can be divided into non-enzymatic and enzymatic [17]. The non-enzymatic antioxidant system comprises ascorbic acid, vitamin E, and flavonoids. The enzymatic antioxidant system includes a series of antioxidant enzymes such as peroxiredoxin (PRX), superoxide dismutase (SOD), catalase (CAT), ascorbate peroxidase (APX), glutathione peroxidase (GPX), and peroxidase (POD) [18], which collectively scavenge ROS [19,20]. Among them, SOD catalyzes the dismutation of O_2_^−^ into H_2_O_2_ and O_2_, after which POD, APX, and PRX further decompose hydrogen peroxide into water and other non-toxic substances [21]. Under ROS-induced stress, the enhancement of antioxidant capacity in plants is attributed to the upregulated expression of a series of ROS-scavenging genes.

*Salix linearistipularis* (*S. linearistipularis*) is a deciduous, clump-forming shrub or small tree belonging to the genus *Salix* of the family Salicaceae. It is mainly distributed in Northeast China, Inner Mongolia, Hebei, Shaanxi, and regions of Russia [22]. *S. linearistipularis* is a dioecious and unisexual flowering plant, which blooms either before or simultaneously with the emergence of leaves. This species is characterized by its strong reproductive capacity, high tolerance to cold, and notable flexibility. It is commonly utilized as a tree species for improving saline-alkali soils [23,24]. *S. linearistipularis* exhibits a strong adaptability to saline-alkali conditions and contributes to soil improvement to a certain extent. Its rhizosphere effect is manifested as a significant increase in soil enzyme activity, soil organic matter content, total nitrogen content, and ammonium nitrogen content, while effectively reducing soil nitrate nitrogen content and electrical conductivity, along with other physicochemical parameters [25].

Despite increasing evidence that ROS play critical roles in plant reproductive development, the functions and regulatory mechanisms of ROS during pistil development and pollination in woody plants remain poorly understood. In particular, little is known about how ROS dynamics are coordinated with pistil developmental progression and pollen–pistil interactions in *S. linearistipularis*. Therefore, in this study, RNA-Seq was employed to elucidate the major metabolic pathways and identify the key genes involved in pistil development. Physiological measurements combined with histochemical staining were conducted to determine the relationship between ROS dynamics and pistil development and pollination. The findings of this study also offer valuable references for breeding, hybridization, and afforestation programs involving *S. linearistipularis*.

## 2. Results

### 2.1. Morphological Observation and RNA-Seq Revealed Distinct Pistil Developmental Stages in S. linearistipularis

**Figure 1:**
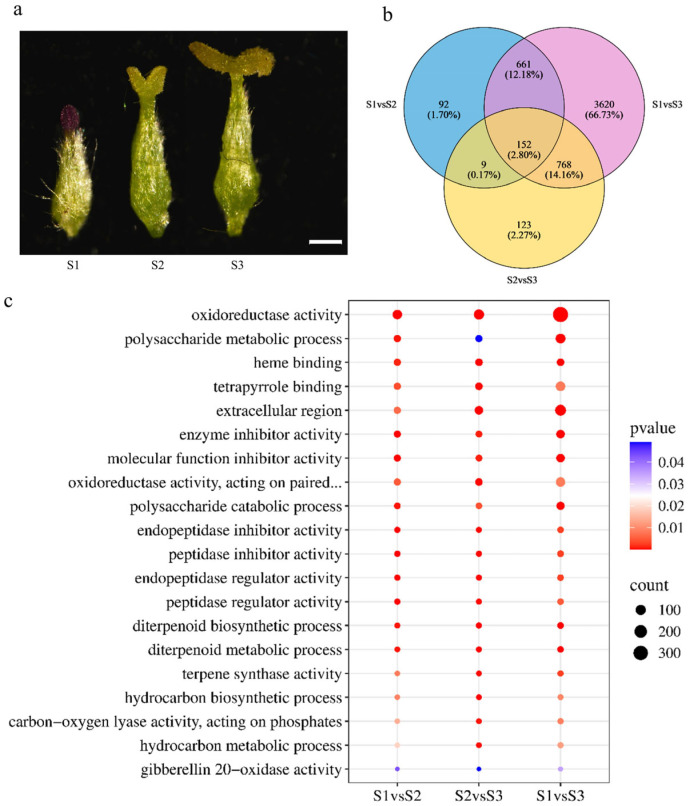
Comprehensive profiling of DEGs and functional enrichment during pistil development in *S. linearistipularis*. (**a**) Pistil developmental morphology in *S. linearistipularis*. From left to right: Stage S1, S2, and S3, respectively. Scale bar = 500 μm; (**b**) Venn diagram of DEGs from each comparison group; (**c**) GO enrichment analysis of DEGs for each comparison group.

Morphological observation revealed that the style and ovary of *S. linearistipularis* pistils are covered with trichomes throughout, and the surface of the stigma is composed of papillary small particles, which function may be associated with pollen recognition and adhesion. At the S1 stage, the pistil length ranged from 2 to 2.6 mm, and the overall structure of the stigma was compact, exhibiting a relatively rounded appearance. The stigma color was predominantly reddish-brown, with a few appearing green. At the S2 stage, the pistil length ranged from 2.5 to 3.0 mm, and the characteristics of stigma division began to emerge, presenting initial forked morphologies such as a “V”-shaped structure. At the S3 stage, the pistil length ranged from 2.9 to 3.5 mm, and the stigma formed a distinct and deeper bifurcation, with the bifurcation angle reaching nearly 180°. Throughout pistil development, the style and ovary remained green. During the S2 and S3 stages, the stigma appeared green, while a few appeared reddish-brown (Figure 1a).

To investigate the regulatory relationship between different developmental stages of the pistil and ROS in *S. linearistipularis*, RNA-Seq was performed on pistils at three developmental stages. Nine RNA samples were sequenced using the Illumina platform. The resulting GC content ranged from 44 to 45%, with Q30 base percentages exceeding 96%, and the error rate ranged from 0.0117 to 0.0118% (Supplementary Table S1). These results indicate that the sequencing data were of high quality and suitable for further analysis. A Venn diagram was constructed to illustrate the distribution of differentially expressed genes among comparison groups, revealing a total of 152 genes involved in pistil development of *S. linearistipularis* (Figure 1b). Based on the transcriptome dataset, 1249 transcription factors were identified and classified into 20 distinct families. Among them, the MYBsuperfamily (216 genes), AP2/ERF (119 genes), and NAC (102 genes) families were the most abundant (Supplementary Figure S1a).

For enrichment analysis, three GO comparison groups (S1 vs. S2, S2 vs. S3, and S1 vs. S3), 20 enriched pathways were selected and integrated based on their count values, which were used for ranking. Among these 20 pathways, several ROS-related pathways were consistently enriched across all three comparison groups, such as oxidoreductase activity, oxidoreductase activity acting on paired donors, with incorporation or reduction in molecular oxygen (Figure 1c). In addition, the top 20 pathways with a *p* value ≤ 0.05 were selected for Kyoto Encyclopedia of Genes and Genomes (KEGG) enrichment analysis. The results showed that enriched pathways included motor proteins, DNA replication, pentose and glucuronate interconversions, homologous recombination, flavonoid biosynthesis, phenylpropanoid biosynthesis, and tryptophan metabolism, among others (Supplementary Figure S1b–d).

### 2.2. The ROS-Related Genes Are Differentially Expressed During Pistil Development in S. linearistipularis

**Figure 2:**
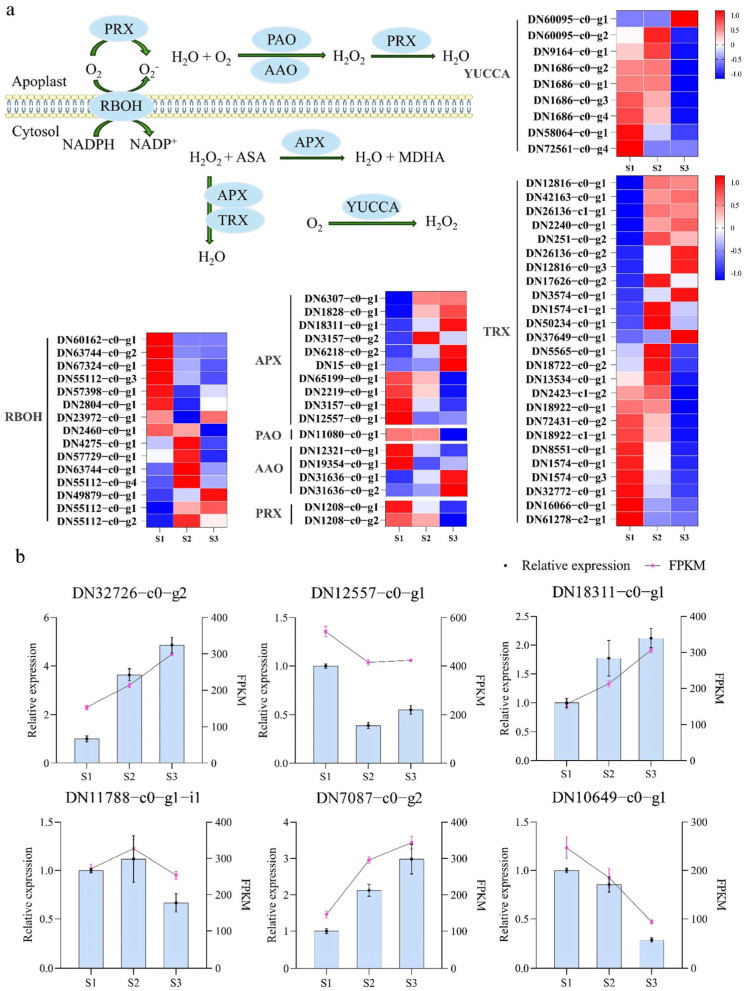
Differential expression of genes associated with ROS production and scavenging in the cytosol. (**a**) Heatmap of gene expression patterns; (**b**) qRT-PCR validation of transcriptomic data. (Data were normalized to the S1 stage set as 1).

To further explore the role of ROS during pistil development of *S. linearistipularis*, genes related to ROS metabolism in the cytosol and apoplast were predicted and analyzed. A total of 15 respiratory burst oxidase homolog (RBOH) genes were found to be differentially expressed, with seven genes showing high expression at the S1 stage, five at the S2 stage, and three at the S3 stage. In addition, ten ascorbate peroxidase (APX) genes were identified, among which four were highly expressed at the S1 stage, while the remaining six exhibited higher expression at the S2 and S3 stages. Both polyamine oxidase (PAO) and peroxiredoxin (PRX) genes were highly expressed at the S1 stage, and four ascorbate oxidase (AAO) genes were detected, with 2 showing high expression at the S1 stage and the other two at the S3 stage. Among nine genes encoding YUCCA monooxygenase (YUCCA) enzymes, only one was highly expressed at the S3 stage. Furthermore, 12 thioredoxin (TRX) genes were upregulated at the S2 and S3 stages, whereas 13 TRX genes showed the lowest expression levels at the S3 stage (Figure 2a). These results indicate dynamic regulation of ROS-generating and ROS-scavenging systems across pistil development, suggesting stage-specific roles for redox modulation.

According to the KEGG enrichment analysis, the flavonoid biosynthesis pathway was among the most significantly enriched pathways. A total of nine genes associated with this pathway were identified, most of which showed high expression at the S1 stage (Supplementary Figure S1e).

In summary, genes related to ROS and flavonoid metabolism exhibited stage-specific expression patterns during pistil development in *S. linearistipularis*, suggesting that the ROS metabolic network plays an important regulatory role across developmental stages. To further validate the reliability of the transcriptome results, several key genes involved in ROS and flavonoid-related pathways were selected for qPCR analysis, and their expression trends were consistent with the RNA-seq data (Figure 2b).

### 2.3. ROS Scavenging Activity Increased During Pistil Development in S. linearistipularis

**Figure 3:**
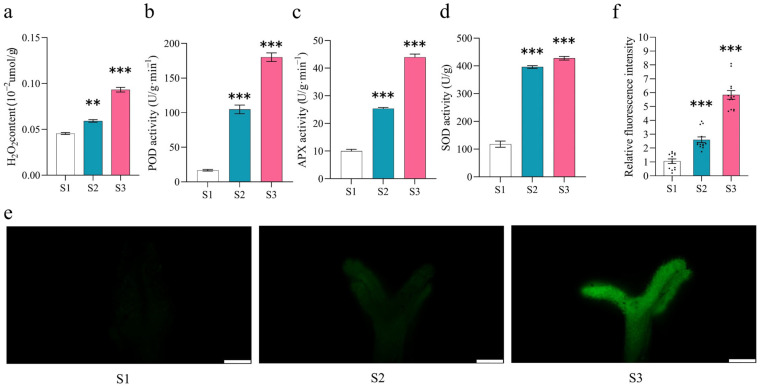
Analysis of ROS accumulation and scavenging activity during pistil development in *S. linearistipularis*. (**a**) H_2_O_2_ content; (**b**) SOD activity; (**c**) POD activity; and (**d**) APX activity. Data are presented as the mean ± SEM (n = 3 biological replicates). Statistical significance was determined by one-way ANOVA with Tukey’s HSD (*** *p* < 0.001, ** *p* < 0.01); (**e**) Measurement of H_2_DCFDA fluorescence intensity in pistils at three developmental stages (S1, S2, and S3). Scale bar = 200 μm; (**f**) Relative ROS levels in pistils across developmental stages. n at S1 = 12; n at S2 = 13; n at S3 = 13. Data were normalized to the S1 stage set as 1. Data are presented as the mean ± SEM (n = 12–13 biological replicates). Statistical significance was determined by one-way ANOVA with Tukey’s HSD (*** *p* < 0.001).

Physiological parameters, including POD, SOD, APX activities, and H_2_O_2_ content, were measured during S1, S2, and S3 stages of pistil development in *S. linearistipularis*. The results showed that from S1 to S3, both antioxidant enzyme activities and the H_2_O_2_ content increased (Figure 3a–d). The ROS accumulation in the stigmas at S1, S2, and S3 stages was measured using H_2_DCF-DA staining. The fluorescence intensity at the S2 stage was 2.5-fold that of the S1 stage, and at the S3 stage was 5.5-fold that of the S1 stage (Figure 3e). These results suggest a crucial role of ROS in the pistil development of *S. linearistipularis*.

### 2.4. ROS Levels Decrease After Pollination and Enhances Pollen Tube Growth in Pistils of S. linearistipularis

**Figure 4:**
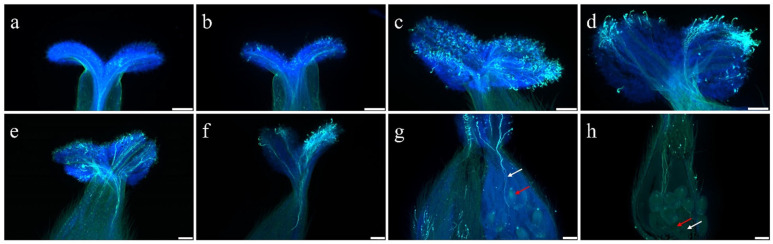
Changes in pollen tube length during 0–12 h after pollination. (**a**) 0 h after pollination. Scale bar = 200 μm; (**b**) 1 h after pollination. Scale bar = 200 μm; (**c**) 3 h after pollination. Scale bar = 200 μm; (**d**) 4 h after pollination. Scale bar = 200 μm; (**e**) 6 h after pollination. Scale bar = 200 μm; (**f**) 8 h after pollination. Scale bar = 200 μm; (**g**) 10 h after pollination. Scale bar = 200 μm; (**h**) 12 h after pollination. Scale bar = 200 μm. White arrows indicate pollen tubes, whereas red arrows indicate the ovary.

To determine whether pollination could reduce the ROS content in pistils and whether ROS levels are associated with pollen tube growth in the pistil, *S. linearistipularis* pollen was evenly applied to the stigmas using a pollination brush, and the ROS fluorescence intensity was observed at 0 h, 0.5 h, 1 h, and 2 h after pollination (Supplementary Figure S2a). The fluorescence intensity at 0 h after pollination was set to 1, and a significant reduction was observed 1.85 times lower at 0.5 h, three times lower at 1 h, and 10.75 times lower at 2 h compared to control (0 h) (Figure 4a–d and Supplementary Figure S2b). The ROS level in the pistil gradually decreased after pollination, with fluorescence intensity becoming extremely weak at 2 h. These results indicate that ROS levels decreases after pollination, and may be associated with pollen germination and pollen tube growth.

Concurrently, we tracked pollen germination and tube growth from 0 to 12 h post-pollination. Germination was negligible within the first 0.5 h but began by 1 h, with an average tube length of 39 μm. Tubes elongated toward the style by 4 h (309 μm) and continued extending toward the ovary through 6–8 h. By 10 h, pollen tubes reached the ovary, and fertilization completed by 12 h. This timeline revealed that approximately 12 h are required for pollen tubes to progress from germination to fertilization in *S. linearistipularis*. Quantitative analysis further showed that growth was not linear, a relatively high elongation rate occurred between 1 and 3 h, but the most substantial increase in length approximately 800 μm, representing that the majority of total elongation took place between 10 and 12 h (Figure 4e–h and Supplementary Figure S2c). This late-stage surge is likely of particular biological significance. Together, these results demonstrate that pollination triggers a sharp decrease in pistil ROS, which appears temporally correlated with subsequent phases of pollen tube elongation and fertilization.

### 2.5. Increase in Stigmatic ROS Inhibits Pollen Tube Germination and Elongation

**Figure 5:**
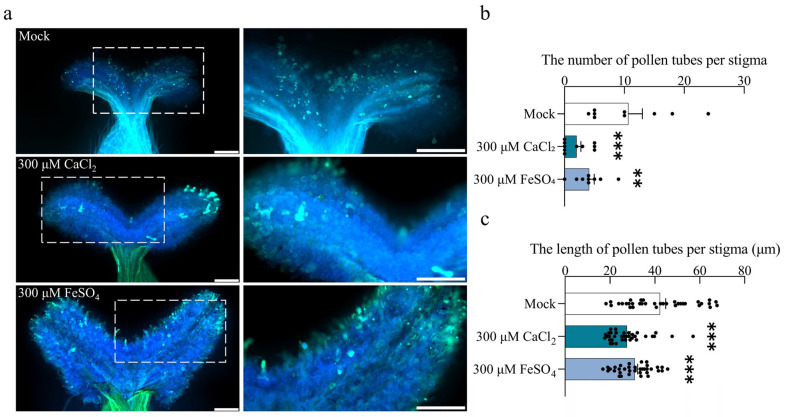
The increase in ROS levels in the stigma inhibits pollen germination and tube growth. (**a**) Effects of 1 h pretreatment of pistils with CaCl_2_ or FeSO_4_ on pollination. (**Left**) 5× magnification. (**Right**) 10× magnification of the boxed area. Scale bar = 200 μm; (**b**) The number of pollen tubes per stigma. Sample sizes were n = 9 (Mock), n = 10 (300 μM CaCl_2_), and n = 9 (300 μM FeSO_4_); (**c**) The length of pollen tubes per stigma. n = 35 per group. Asterisks above the bars indicate the statistical significance of differences compared to the Mock group. Data are presented as the mean ± SEM. Statistical significance was determined by one-way ANOVA with Tukey’s HSD (*** *p* < 0.001, ** *p* < 0.01).

To investigate the relationship between ROS levels and pollen tube germination, we treated *S. linearistipularis* pistils with 300 µM calcium chloride (CaCl_2_) or ferrous sulfate (FeSO_4_) and control (mock) for 1 h. This treatment significantly increased stigma ROS levels compared to the mock (Supplementary Figure S3a,b). When these treated stigmas were subsequently pollinated and incubated for one hour, pollen tube germination and growth were markedly inhibited. The number of pollen tubes on the stigma was reduced by approximately 6-fold in the CaCl_2_ group and 3-fold in the FeSO_4_ group compared to the mock (Figure 5a,b). Furthermore, the average pollen tube length in both treatment groups was significantly shorter than in the control (Figure 5c). Collectively, these results demonstrate that elevated ROS levels significantly inhibit pollen tube germination and elongation on the stigma.

### 2.6. Reduced ROS Levels Promote Pollen Tube Growth

**Figure 6:**
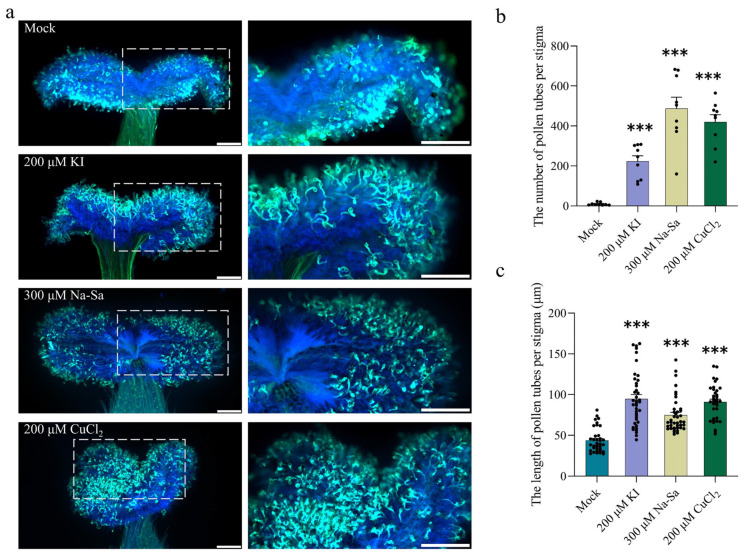
The decrease in ROS levels in the stigma promotes pollen germination. (**a**) Effects of 1 h pretreatment of pistils with potassium iodide (KI), sodium salicylate (Na-SA), and copper chloride (CuCl_2_) on pollination. (**Left**) 5× magnification. (**Right**) 10× magnification of the boxed area. Scale bar = 200 μm; (**b**) The number of pollen tubes per stigma. Each group represents 12 biological replicates; (**c**) The length of pollen tubes per stigma. Sample sizes were n = 35 (Mock), n = 40 (200 μM KI), n = 40 (300 μM Na-SA), and n = 36 (200 μM CuCl_2_). Asterisks above the bars indicate the statistical significance of differences compared to the Mock group. Data are presented as the mean ± SEM. Statistical significance was determined by one-way ANOVA with Tukey’s HSD (*** *p* < 0.001).

To test whether reduced ROS enhances pollen tube growth, pistils were treated for 1 h with chemical agents known to interfere with ROS. The ROS inhibitor potassium iodide (KI; 200 µM), the ROS scavenger sodium salicylate (Na-SA; 300 µM), and copper(II) chloride (CuCl_2_; 50 µM). Fluorescence intensity of ROS in the stigma was then measured using the probe H_2_DCF-DA. All three treatments significantly reduced ROS levels compared to the mock control (Supplementary Figure S3c). CuCl_2_ showed the strongest inhibitory effect, reducing ROS fluorescence to approximately one-sixth of the mock level (Supplementary Figure S3d). Following the 1 h pretreatment, stigmas were hand-pollinated uniformly using a fine brush (Figure 6a). Pollen performance was assessed by counting pollen tubes and measuring their lengths. The 300 µM Na-SA treatment resulted in the highest number of pollen tubes, averaging 487, which was approximately 58 times greater than the mock control (Figure 6b). In terms of elongation, the mock group produced pollen tubes with an average length of about 40 µm. All treatments promoted longer pollen tubes, with the 200 µM KI treatment being most effective, yielding an average pollen tube length of 95 µm (Figure 6c). Together, these results indicate that pharmacologically reducing stigma ROS levels significantly enhances both pollen germination and tube elongation in *S. linearistipularis*.

### 2.7. Inhibition of RBOH-Mediated ROS Production Promotes Pollen Tube Germination

**Figure 7:**
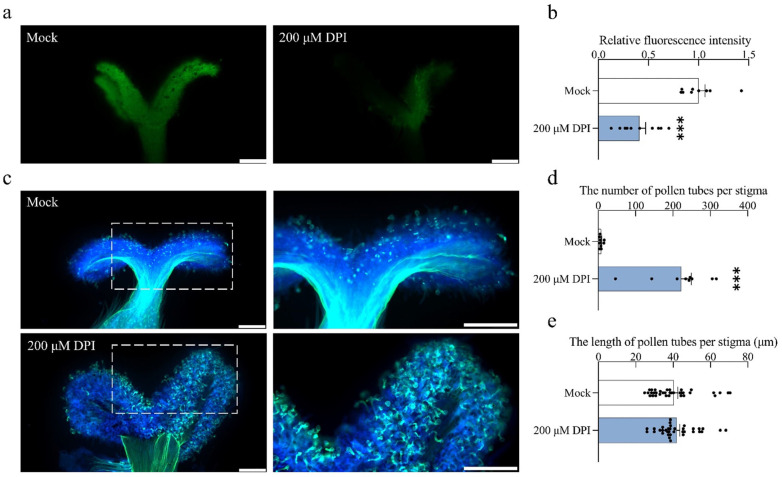
Effect of ROS on pollen germination under DPI treatment. (**a**) ROS levels in stigmas after 1 h diphenyleneiodonium chloride (DPI) treatment; (**b**) Relative fluorescence intensity. Sample sizes were n = 9 (Mock), n = 10 (200 μM DPI). Data were normalized to the Mock set as 1; (**c**) Effects of 1 h pretreatment of pistils with DPI on pollination. (**Left**) 5× magnification. (**Right**) 10× magnification of the boxed area; (**d**) The number of pollen tubes per stigma. n = 9 per group; (**e**) The length of pollen tubes per stigma. n = 35 per group. Asterisks above the bars indicate the statistical significance level (ns, not significant). ns comparisons are omitted for clarity. Data are presented as the mean ± SEM. Statistical significance was determined by one-way ANOVA with Tukey’s HSD (*** *p* < 0.001). Scale bar = 200 μm.

To further substantiate the ROS function in pollen germination and tube growth, a plant-specific NADPH oxidase, RBOH whose primary function is to catalyze the production of ROS, mainly superoxide anion (O_2_^−^) was inhibited using diphenyleneiodonium (DPI). The DPI was applied to *S. linearistipularis* pistils for 1 h and manually pollinated. The results revealed that ROS fluorescence intensity decreased to 40% compared to the mock (Figure 7a,b). At 1 h after pollination, the number of pollen tubes in the 200 μM DPI treatment group was 30 times that of the mock (Figure 7c,d). In contrast, the pollen tube length showed no significant difference compared with the mock group (Figure 7e). These results showed that the inhibition of RBOH activity reduced stigmatic ROS levels, and affects pollen germination, rather than elongation.

## 3. Discussion

*S. linearistipularis* not only exhibits remarkable tolerance to highly alkaline soils (pH > 9) [26] but also has the capacity to significantly ameliorate soil salinization and alkalization [25]. As one of the few plant species capable of thriving in saline–alkaline environments [27], *S. linearistipularis* serves as a valuable biological resource for studying plant adaptation to extreme soil conditions. This study addresses a key gap in the reproductive biology of *S. linearistipularis* by elucidating the role of ROS in pistil development and pollination. By revealing how ROS homeostasis influences pollen germination and pollen tube growth, our findings provide a physiological basis for understanding reproductive efficiency in this species, which contribute to population breeding and the sustainable cultivation of *S. linearistipularis* in saline–alkaline soils.

RNA-Seq technology has driven a surge in research integrating transcriptomic analysis with studies of pistil development and stigma pollination responses, significantly clarifying the molecular networks and underlying mechanisms regulating plant reproductive development [28,29,30]. However, research on pistil development and pollination in *S. linearistipularis* remains limited. In the present study, several pathways were enriched in the three comparison groups (S1 vs. S2, S2 vs. S3, and S1 vs. S3), including oxidoreductase activity, flavonoid biosynthesis, and phenylpropanoid biosynthesis (Figure 1c). These pathways are central to regulating the oxidative environment and cell wall composition, both critical factors in pollen–pistil interactions. To manage the cytotoxic effects of reactive oxygen species (ROS), plants employ efficient enzymatic antioxidant systems [31]. The cytoplasm is a primary hub for ROS signaling, integrating signals from extracellular and organelle sources while housing enzymatic systems that fine-tune ROS levels to modulate developmental signaling [32]. For example, the membrane-bound NADPH oxidases generate extracellular ROS by catalyzing the formation of O_2_^−^ from O_2_, a process dependent on cytosolic NADPH as an electron donor [33,34]. In the *Arabidopsis nadp-me2* mutant, the loss of NADP-ME2, an enzyme catalyzing the synthesis of NADPH, results in a significant decrease in intracellular NADPH levels and subsequently leads to a marked reduction in ROS production [35,36]. This finding provides direct evidence that NADPH, as an electron donor, plays an essential role in cytosolic RBOH-mediated ROS generation. In our data, genes encoding key antioxidant enzymes, like ascorbate peroxidase (APX) and peroxidases (PRX), showed distinct expression patterns. APX genes maintained high expression throughout all stages, with peaks in S1 and S3, while PRX expression was significantly elevated specifically at the S1 stage (Figure 2a). APX and PRX are vital for cellular protection under stress [37,38], with documented roles in enhancing drought and salt tolerance [39,40]. In the context of pollination, the precise spatiotemporal regulation of these antioxidants is crucial for maintaining ROS homeostasis, which directly influences pollen tube growth and guidance by preventing cytotoxic damage while preserving necessary signaling gradients.

Flavonoids are a class of plant secondary metabolites that include coumarins, flavones, flavonols, isoflavones, dihydroflavonols, flavanols, and anthocyanins [41,42]. Flavonoids not only play important roles in plant responses to biotic and abiotic stresses but also possess strong antioxidant activities. Flavanols, characterized by their hydroxylated phenolic ring structures, act as electron acceptors, thereby functioning in the scavenging of ROS [43,44,45]. Moreover, flavanols promote pollen development and pollen tube germination by reducing ROS accumulation [46]. Other phenylpropanoid-derived compounds, such as flavonoids, lignins, and coumarins, also serve as highly effective antioxidants [47]. Flavonoid metabolism represents a major branch of phenylpropanoid metabolism, leading to the formation of one of the largest groups of polyphenolic metabolites in plants [48]. In addition, lignin plays essential roles in anther development, defense against pathogen invasion, and adaptation to abiotic stresses [49]. Lignin deposition also contributes to stylar and transmitting tract architecture, which physically guides the pollen tube toward the ovule. Therefore, the co-enrichment of antioxidant (oxidoreductase, APX/PRX) and specialized metabolite (flavonoid/phenylpropanoid) pathways indicates a sophisticated, dual-layered system in *S. linearistipularis*. This system likely functions to manage oxidative stress in saline–alkaline soils while simultaneously facilitating the precise ROS modulation and structural support required for successful pollination and pollen tube germination.

The level of ROS increases with the enhancement of metabolic activity. During this process, the plant’s antioxidant system dynamically adjusts to maintain ROS homeostasis, which is essential for normal plant development [12]. However, excessive accumulation of ROS beyond the scavenging capacity of the antioxidant system can lead to oxidative damage [50]. The antioxidant system in plants consists of both enzymatic and non-enzymatic components [51,52]. The enzymatic antioxidant system includes CAT, SOD, APX, and POD, whereas the non-enzymatic antioxidant system comprises ascorbic acid, anthocyanins, carotenoids, vitamin E, and flavonoids. As one of the major ROS, H_2_O_2_ functions as a signaling molecule at low concentrations but exhibits cytotoxic effects at high levels [53]. Physiological measurements in this study showed that from S1 to S3, both H_2_O_2_ content and antioxidant enzyme activities increased concurrently (Figure 3a–d). Maria et al. found that the expression level of Cu/Zn SOD was relatively higher in the later stages of pistil development than in the early stages [54]. Podobedova et al. reported that the H_2_O_2_ content increased in the pollen of SOD-transformed plants [55]. This suggests that during pistil development, metabolic activity in the S1 and S2 stages remains relatively low, and the stigmas at these stages may not yet possess strong pollination capability, resulting in limited ROS production and a reduced demand for antioxidant protection. At the S3 stage, however, enhanced metabolic activity likely corresponds to the optimal phase for pollination, requiring stronger antioxidant capacity. Consequently, both antioxidant enzyme activities and ROS levels were significantly higher in S3 than in S1 and S2 (Figure 3e). These findings indicate that the pistil actively regulates its redox state, possibly preparing for the subsequent pollination process.

The cytotoxic and signaling roles of ROS are mediated by their intracellular concentration, where elevated levels induce cellular damage while lower levels are essential for signaling [56,57]. In *S. linearistipularis*, excessively high ROS levels in the stigma inhibited pollen germination, whereas maintaining ROS within a lower, optimal range, through treatment with ROS inhibitors, significantly promoted it. This explains why, even though ROS levels in the 200 μM CuCl_2_ treatment were lower than in the 300 μM Na-SA and 200 μM KI treatments, the 300 μM Na-SA treatment exhibited the strongest promotive effect (Figure 5, Figure 6 and Figure 7 and Supplementary Figure S3). These results indicate that stigma ROS in *S. linearistipularis* operate within an optimal concentration window, where excessive ROS are inhibitory, while moderate, controlled levels facilitate pollen germination. This concentration-dependent dual role of ROS is not uncommon in plant physiology. For instance, Tian et al. showed that in *Arabidopsis thaliana*, inhibiting RBOH with DPI to deplete endogenous H_2_O_2_ nearly abolished brassinosteroid-induced hypocotyl elongation. When exogenous H_2_O_2_ was applied, hypocotyl elongation increased with H_2_O_2_ concentrations up to 0.75 mM, but the promoting effect diminished at 1 mM [58].

Interestingly, studies have shown that in *Arabidopsis thaliana* and many other angiosperms, the stigma surface typically maintains a high level of ROS, which significantly decreases once pollen successfully adheres to the stigma surface [59]. This pattern is consistent with the trend observed in *S. linearistipularis*, where ROS levels markedly declined within 0.5 h after pollination (Supplementary Figure S2a,b). The observation that pollen germination was promoted when the ROS level in the stigma was maintained at a relatively low range by inhibitor treatment suggests that such a condition may mimic the ROS environment of a successfully pollinated stigma.

Collectively, by integrating transcriptomic, physiological, and biochemical analyses, this study demonstrates that ROS levels are dynamically regulated during pistil development and pollination in *S. linearistipularis*, and ROS homeostasis is closely associated with pollen germination and pollen tube growth. Importantly, experimental investigation of ROS levels revealed the existence of an optimal ROS range required for successful fertilization. Taken together, these findings confirm that ROS function as critical signaling molecules regulating sexual reproduction in *S. linearistipularis* and provide a physiological and molecular basis for future breeding studies.

## 4. Materials and Methods

### 4.1. Collection of Experimental Materials and Transcriptome Sequencing

The pistils of *S. linearistipularis* were collected from the experimental base of Northeast Forestry University in Anda City, Heilongjiang Province (125°22′ E, 46°27′ N). Pistil samples were collected from approximately twelve trees, each about 15 years old. The developmental stages of the *S. linearistipularis* pistil were divided into three periods. In the early stage (S1), the stigma presents relatively intact and undivided small protrusions. In the mid-stage (S2), the stigma begins to show signs of splitting, presenting initial forked shapes, such as a “V”-shaped structure. In the later stage (S3), the degree of stigma splitting further deepens, with some stigmas reaching nearly 180° in bifurcation angle. Three independent biological replicates were collected for each stage. Each biological replicate consisted of 0.3–0.5 g of pistil tissue, corresponding to approximately 2000 pistils at S1, 1500 at S2, and 1200 at S3. This sampling procedure was performed three separate times, with each replicate prepared individually. All samples were rapidly frozen in liquid nitrogen and stored at −80 °C.

RNA was extracted using the Omega Plant RNA Extraction Kit (R6628; Bio-Tek, Winooski, VT, USA), following the protocol outlined in the kit’s manual. After extraction, RNA quality and concentration were assessed using agarose gel electrophoresis and Ultraviolet–Visible spectrophotometry (Nanodrop 2000; Thermo Fisher Scientific, Waltham, MA, USA). RNA-Seq and RNA library construction were performed by Shanghai Meiji Biological Medicine Technology Company Limited (Shanghai, China). Nine RNA samples (three replicates in each period) were sequenced using the Illumina platform. Differential gene expression analysis between groups was conducted based on expression quantification results, identifying genes with differential expression between the two groups. The differential analysis software was DESeq2 3.22, and the screening thresholds were: |log2FC| > =1.0 and FDR < 0.05. Transcriptome data analysis was performed using the platform (https://www.majorbio.com/; accessed on 1 October 2024).

### 4.2. Physiological Parameter Measurement

Peroxidase (POD) activity was measured using the guaiacol method, superoxide dismutase (SOD) activity was assessed using the nitroblue tetrazolium (NBT) method, ascorbate peroxidase (APX) activity was determined using the ascorbate method, and hydrogen peroxide (H_2_O_2_) content was measured using a colorimetric assay. Enzyme activity measurements were performed using a multifunctional microplate reader.

### 4.3. Detection of ROS in the Pistil

ROS levels in pistils were detected using 2′,7′-Dichlorodihydrofluorescein Diacetate (H_2_DCF-DA) as a fluorescent probe. Pistils were immersed in phosphate-buffered saline (PBS) buffer containing 20 μM H_2_DCF-DA and incubated at 4 °C for 1 h, followed by repeated washing with PBS buffer before observation. All samples were imaged using Leica DM 4 B fluorescence microscope (Wetzlar, Germany).

### 4.4. In Vitro Culture Experiment of Pistil

Pollen germination medium (PGM) consisted of 10% sucrose, 5 mM CaCl_2_, 5 mM KCl, 1 mM MgSO_4_·7H_2_O, and 0.01% H_3_BO_3_, with the PH adjusted to 7.5. After sterilization, the medium was stored at 4 °C. Before use, 1% agarose was added to prepare solid medium. Stock solutions of 200 mM KI, 200 mM Na-SA, 200 mM CuCl_2_, 10 mM DPI, and 200 mM CaCl_2_ were prepared in advance, while 300 μM FeSO_4_ was freshly prepared prior to use. Before use, the stock solutions were added to the solid germination medium and diluted to the desired working concentrations, while the control group received an equal volume of deionized water. The pistils were then placed in PGM and incubated for 1 h in an illuminated, constant-temperature growth chamber at 26 °C and 40% humidity. *S. linearistipularis* pollen was evenly applied onto the stigma using a pollination brush, followed by continued incubation under the same conditions for a defined period. Finally, the pistils were stored in fixative solution and stained with aniline blue for microscopic observation of pollen tube germination. The chemical reagents used—KI, Na-SA, CuCl_2_, DPI—were all preliminarily tested at two concentrations (200 μM and 300 μM). The data shown in the subsequent figures are the optimal concentrations at which each reagent exhibits the most significant effect in the said determination. Complete datasets for all concentrations are provided in the Supplementary Tables S6–S14.

### 4.5. Fluorescence Microscopy Observation

We collected fresh pistil samples and placed them in a solution of anhydrous ethanol: glacial acetic acid in a ratio of 3:1, then stored at 4 °C. Before aniline blue staining, the pistil samples were rinsed three times with deionized water. Subsequently, samples were softened in 1 M NaOH at 65 °C for 45 min in an incubator. After softening, residual NaOH on the sample was removed by washing. The staining solution was prepared by dissolving 0.1% water-soluble aniline blue in 0.1 M K_3_PO_4_·3H_2_O buffer and aged at 4 °C for 3 days before use. The pistils were then stained in the aniline blue solution overnight at 4 °C in the dark. The attachment and germination of pollen were observed using Leica DM 4 B fluorescence microscope (Wetzlar, Germany).

### 4.6. Data Analysis

The data, such as fluorescence quantification, physiological parameter measurement, ROS fluorescence intensity, the number of pollen tubes, and the length of pollen tubes, were all represented as bar graphs generated by GraphPad Prism 9.5.0. The detection of ROS fluorescence intensity, measurement of pollen tube length and scale bar were all accomplished using the software image J 1.52a. Each dot in the bar graphs represents an individual data point used for statistical analysis, and error bars indicate the mean ± standard error of the mean (SEM). One-way ANOVA with Tukey’s HSD test was used to assess significant differences among groups. Significance levels are indicated above the bars as follows: * *p* < 0.05, ** *p* < 0.01, *** *p* < 0.001.

## Data Availability

Data will be made available on request.

## Supplementary Materials

The following supporting information can be downloaded at: https://www.mdpi.com/article/10.3390/plants15010168/s1, Figure S1: Transcriptomic analysis of nine pistil samples across developmental stages; Figure S2: Changes in fluorescence intensity and pollen tube length after pollination; Figure S3: Effects of different drug treatments on stigmatic ROS levels; Figure S4: Morphological characteristics of male and female inflorescences and reproductive status in *Salix linearistipularis*. Table S1: GC Content and Error Rate of 9 RNA samples; Table S2: qRT-PCR Primer Sequence; Table S3: ROS fluorescence intensity during pistil development; Table S4: ROS fluorescence intensity in stigmas after pollination; Table S5: Pollen tube length at 0–12 h after pollination; Table S6: Relative fluorescence intensity of stigma after 1 h treatment with FeSO_4_ and CaCl_2_; Table S7: Number of pollen tubes in the stigma after treatment with FeSO_4_ and CaCl_2_ followed by 1 h pollination; Table S8: Length of pollen tubes in the stigma after treatment with FeSO_4_ and CaCl_2_; Table S9: Relative fluorescence intensity after 1 h ROS inhibitor treatment at various concentrations; Table S10: Number of pollen tubes after treatment with a ROS inhibitor at various concentrations followed by 1 h pollination; Table S11: Length of pollen tubes after treatment with a ROS inhibitor at various concentrations followed by 1 h pollination; Table S12: Relative fluorescence intensity of stigma after 1 h treatment with DPI; Table S13: Number of pollen tubes in the stigma after treatment with DPI followed by 1 h pollination; Table S14: Length of pollen tubes in the stigma after treatment with DPI followed by 1 h pollination.
