# Supplementary material for: Reactive Oxygen Species Homeostasis Regulates Pistil Development and Pollination in Salix linearistipularis
Source: Plants (Basel). 2026 Jan 5;15(1):168. doi: 10.3390/plants15010168 (PMC12787920; doi:10.3390/plants15010168)
Supplement: Supplementary file 1 [file plants-15-00168-s001.zip › supplementary figures.pdf]

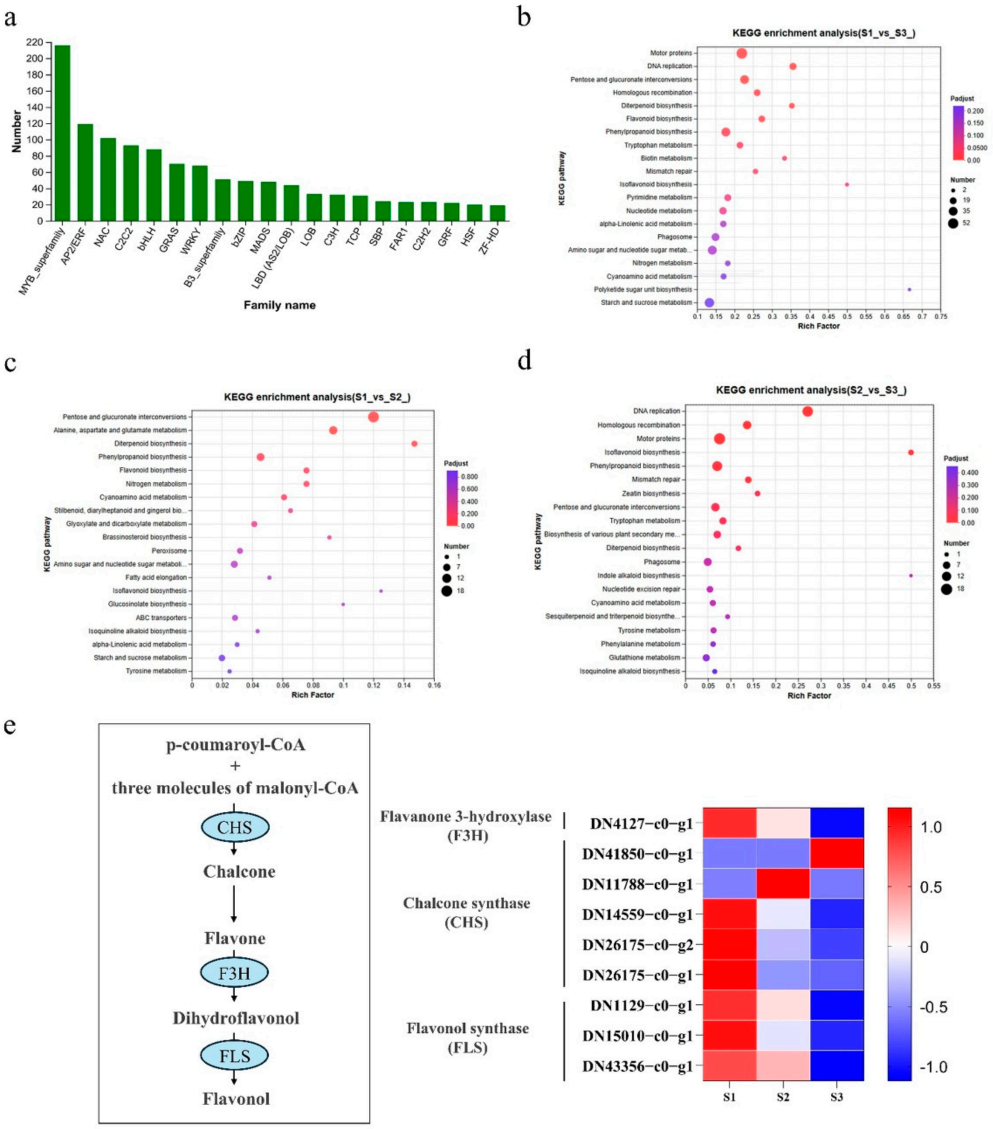

Supplemental Fig.1 Transcriptomic analysis of nine pistil samples across developmental stages. (**a**) Analysis of transcription factor families; (**b**) KEGG enrichment analysis of S1 vs S3; (**c**) KEGG enrichment analysis of S1 vs S2; (**d**) KEGG enrichment analysis of S2 vs S3; (**e**) Heatmap analysis of genes related to the flavonoid biosynthesis pathway.

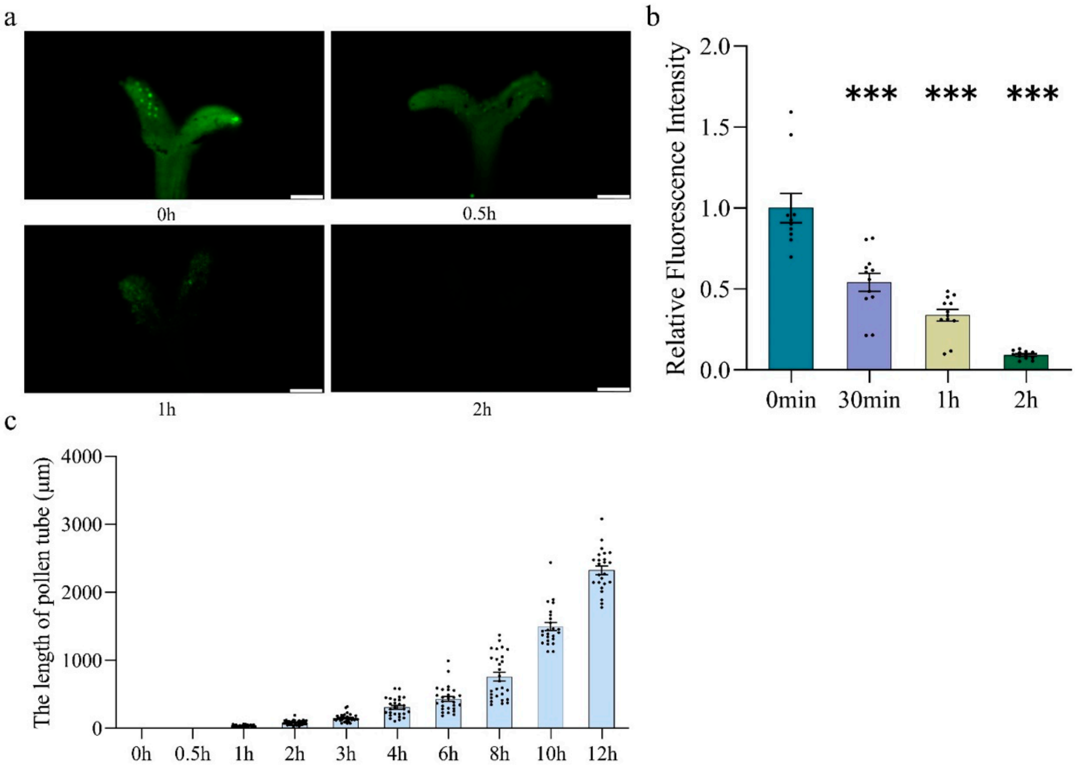

Supplemental Fig.2 Changes in fluorescence intensity and pollen tube length after pollination. (**a**) Measurement of H2DCFDA fluorescence intensity in pistils after pollination. Scale bar = 200 μm; (**b**) Relative ROS levels in stigmas after pollination. Sample sizes were n = 10 (0min), n = 12 (30min), n = 12 (1h), and n = 12 (2h). Data were normalized to the 0h stage set as 1; (**c**) Quantification of pollen tube length at various time points after pollination. Sample sizes were n = 32 (1h), n = 35 (2h), n = 33 (3h), n = 27 (4h), n = 27 (6h), n = 28 (8h), n = 24 (10h) and n = 24 (12h). Asterisks above the bars indicate the statistical significance of differences compared to the Mock group. Data are presented as the mean ± SEM. Statistical significance was determined by one-way ANOVA with Tukey's HSD (***$p < 0.001$).

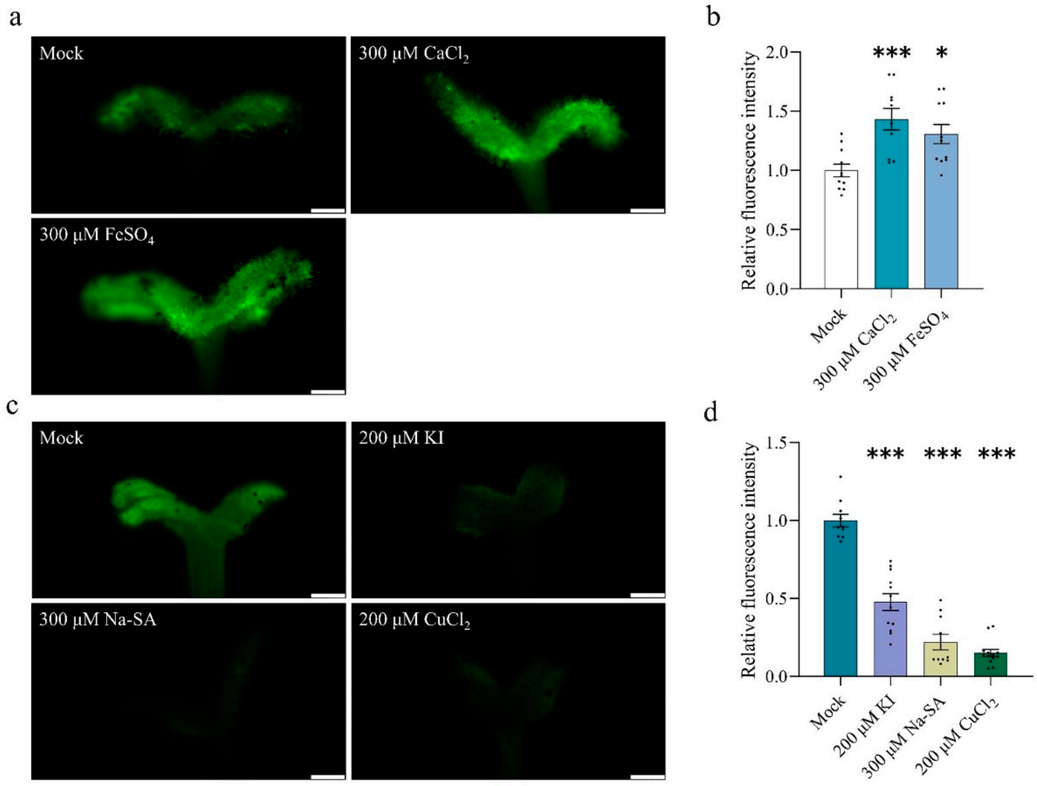

Supplemental Fig.3 Effects of different drug treatments on stigmatic ROS levels. (**a**) ROS levels in stigmas treated with $FeSO_4$ and $CaCl_2$; (**b**) Relative fluorescence intensity. Sample sizes were n = 11 (Mock), n = 10 (300 µM $CaCl_2$), and n = 11 (300 µM $FeSO_4$); (**c**) ROS levels in stigmas treated with KI, Na-SA, and $CuCl_2$; (**d**) Relative fluorescence intensity. Sample sizes were n = 10 (Mock), n = 12 (200 µM KI), n = 10 (300 µM Na-SA), and n = 13 (200 µM $CuCl_2$). Data were normalized to the Mock set as 1. Asterisks above the bars indicate the statistical significance of differences compared to the Mock group. Data are presented as the mean ± SEM. Statistical significance was determined by one-way ANOVA with Tukey's HSD (***$p < 0.001$, *$p < 0.05$.) Scale bar = 200 µm.

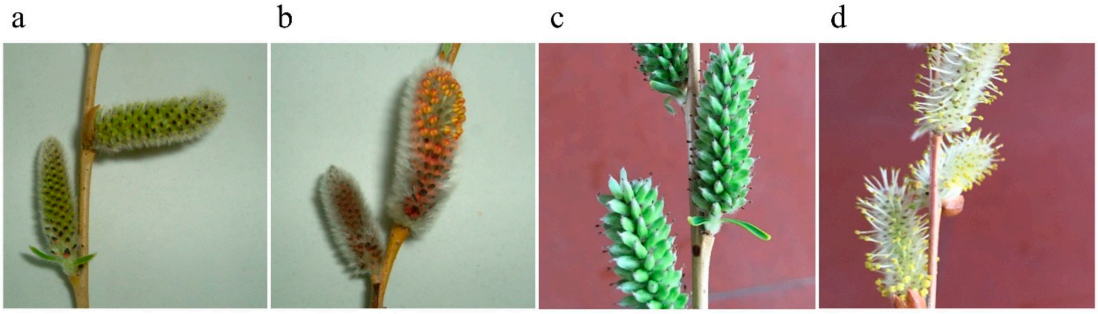

Supplemental Fig.4 Morphological characteristics of male and female inflorescences and reproductive status in Salixleariistipularis. (**a**) Female inflorescences before pollination; (**b**) Male inflorescences; (**c**) Female inflorescences after pollination, exhibiting seed set and fruit development; (**d**) Male inflorescences at the same developmental stage, showing pollen release.
